# Distinct α subunit variations of the hypothalamic GABA_A _receptor triplets (αβγ) are linked to hibernating state in hamsters

**DOI:** 10.1186/1471-2202-11-111

**Published:** 2010-09-06

**Authors:** Raffaella Alò, Ennio Avolio, Anna Di Vito, Antonio Carelli, Rosa Maria Facciolo, Marcello Canonaco

**Affiliations:** 1 Comparative Neuroanatomy Laboratory of Ecology Department, University of Calabria, Ponte Pietro Bucci, 87030 Arcavacata di Rende, Cosenza-Italy

## Abstract

**Background:**

The structural arrangement of the γ-aminobutyric acid type A receptor (GABA_A_R) is known to be crucial for the maintenance of cerebral-dependent homeostatic mechanisms during the promotion of highly adaptive neurophysiological events of the permissive hibernating rodent, i.e the Syrian golden hamster. In this study, *in vitro *quantitative autoradiography and *in situ *hybridization were assessed in major hypothalamic nuclei. Reverse Transcription Reaction-Polymerase chain reaction (RT-PCR) tests were performed for specific GABA_A_R receptor subunit gene primers synthases of non-hibernating (NHIB) and hibernating (HIB) hamsters. Attempts were made to identify the type of αβγ subunit combinations operating during the switching ON/OFF of neuronal activities in some hypothalamic nuclei of hibernators.

**Results:**

Both autoradiography and molecular analysis supplied distinct expression patterns of all α subunits considered as shown by a strong (p < 0.01) prevalence of α_1 _ratio (over total α subunits considered in the present study) in the medial preoptic area (MPOA) and arcuate nucleus (Arc) of NHIBs with respect to HIBs. At the same time α_2 _subunit levels proved to be typical of periventricular nucleus (Pe) and Arc of HIB, while strong α_4 _expression levels were detected during awakening state in the key circadian hypothalamic station, i.e. the suprachiasmatic nucleus (Sch; 60%). Regarding the other two subunits (β and γ), elevated β_3 _and γ_3 _mRNAs levels mostly characterized MPOA of HIBs, while prevalently elevated expression concentrations of the same subunits were also typical of Sch, even though this time during the awakening state. In the case of Arc, notably elevated levels were obtained for β_3 _and γ_2 _during hibernating conditions.

**Conclusion:**

We conclude that different αβγ subunits are operating as major elements either at the onset of torpor or during induction of the arousal state in the Syrian golden hamster. The identification of a brain regional distribution pattern of distinct GABA_A_R subunit combinations may prove to be very useful for highlighting GABAergic mechanisms functioning at least during the different physiological states of hibernators and this may have interesting therapeutic bearings on neurological sleeping disorders.

## Background

Hibernation is a unique physiological condition that permits animals to survive under extraordinary climatic and stressful conditions [1]. This condition has been largely studied on the Syrian golden hamster (*Mesocricetus auratus*), a facultative hibernator (HIB) that displays profound decreases in oxidative metabolism and body temperature during bouts of prolonged torpor interrupted every 5 to 14 days by brief periodic arousals. In such an interval animals spontaneously re-warm to 37°C (euthermic state) for 24-48 hrs [2,3]. Consequently, entering and exiting from torpor requires a notable amount of energy in spite of reduced blood flow, oxygen and glucose delivery as much as 90% of normal value. In addition, a neuroprotective program with adaptive homeostatic mechanisms such as reprogramming of gene expression especially for traumatic fluctuation of cerebral blood flow is activated during these states [4,5]. Although this adaptive physiological condition has fascinated researchers, little is still known about hypothalamic molecular mechanisms regulating hibernation. Recently, interests have been directed to the major cerebral inhibitory neuroreceptor system of mammalian, i.e. γ-aminobutyric acid type A receptor (GABA_A_R) that by operating at a low temperature [6], maintain hypothalamic neuronal activities of HIBs in equilibrium especially during energy balance processes [7].

GABA_A_Rs are members of the cys-loop family of ligand gated ion channels [8] arranged in a pentameric fashion around a central ion channel [9]. At present 20 different classes of subunits and namely α (1-6), β(1-4), γ (1-3), δ, ε, θ, π and ρ (1-3) are combined and assembled to form this highly complex pentameric GABA_A_Rs ionophore molecule [10]. Of these subunits α, β and γ are the most common combinations characterizing GABA_A_R that also determine the overall biophysical and pharmacological properties of this receptor [11]. In particular, it is α subunit that is involved in the assembly of other sequences plus expression of pharmacological functions as shown by α_1,2,4,5 _exhibiting varying degrees of sensitivity to benzodiazepines (BDZ) [12]. Moreover, β and γ subunits also seem to participate with the expression of α subunit as suggested by their constant ratio of 1:1:1 or 1:1:0.5 characterizing most GABA_A_R subunit compositions [13] plus being responsible, as in the case of β_3 _[14] and γ_2 _[15], for the induction of homeostatic, sedative-like and plasticity events. Now, since multiple GABA_A_R subtypes differing in subunit composition, localization and functional properties exist, it may very well be that the various fine-tuning roles of neuronal circuits and genesis of network oscillations [16,17] are predominately linked to α, β and γ combinations. Indeed, specific α-containing GABA_A_R subunits do represent a major facet of homeostatic synaptic plasticity [15]. As a consequence this and the other subunits do appear to contribute to excitatory/inhibitory homeostasis processes of episodic ischemic events typical of both hibernation as well as neurodegenerative disorders [14,15,18].

On the basis of the above considerations, it was our intention to identify the distribution pattern and combination preferences of some specific α (α_1,2,4,5_) along with β (β_2,3_) plus γ (γ_2,3_) subunits in the major hypothalamic regions of HIB and non-HIB (NHIB) states. For such a purpose, the golden hamster resulted to be an adequate model since it undergoes bouts of torpor (3-5 days), which allowed us to examine hypothalamic neuronal features during this physiological state by integrating *in vitro *quantitative autoradiography results to reverse transcription reaction-Polymerase chain reaction (RT-PCR) and *in situ *hybridization data. The correlation of distinct GABA_A_R subunit combinations especially in a region-specific fashion may help to unravel the type of subunits operating during hibernation and this may provide interesting insights regarding their role on neurodegenerative disorders such as ischemia that is typical of arousal state [19].

## Methods

### Animals

For the present study, female sexually mature *Mesocricetus auratus *(100-120 g; Charles River, Italy) were used (n = 21). The hamsters, which had free access to food and water were entrained for one to two days at a temperature of 30°C and to a 12-h light/12-h dark cycle before dividing animals into two groups. A first group (n = 6), defined euthermics (NHIB) consisted of hamsters being maintained under these conditions throughout the entire testing period. The other group (n = 6), which consisted of HIB hamsters were entrained to a temperature 8°C and to a dark local for 20 days. All animals were decapitated and their brains were rapidly removed, frozen using powered dry ice after which stored at -40°C until sectioning at the cryostat and thaw-mounting onto gelatin-coated slides according to previous studies [20] for neuroanatomic and molecular studies.

Animal maintenance and all experimental procedures were carried out in accordance with the Guide for Care and Use of Laboratory Animals issued by the European Communities Council Directive of 24 November 1986 (86/609/EEC). Efforts were made to minimize animal suffering and reduce the number of specimens used.

### In vitro quantitative autoradiography

For this study, a competition binding analysis was performed in order to establish the different pharmacological features of the specific GABA_A_R α subunit radioligand [^3^H] flumazenil (Ro 15-1788) in the major brain region involved with hibernating rhythms and namely the hypothalamus [7]. Briefly, coronal brain sections (2 sections per slide; 12 μm-thick) of HIB and NHIB hamsters were incubated for 1 h at room temperature in 50 mM Tris HCl, pH 7.4, containing 2 nM [^3^H] Ro 15-1788 ± 0.5 μM of the imidazopyridine zolpidem plus different concentrations (500 nM-1 nM) of some agonist and antagonists of GABA_A_R α subunits and namely: the highly selective α_1 _agonist - zolpidem (Synthelabo Recherche, France), the highly selective α_2 _benzodiazepine agonist - flunitrazepam, the highly selective antagonist of α_4 _- the imidazobenzodiazepine Ro 15-4513 and the highly selective inverse agonist of α_5 _- Ry 080 (kindly provided by Dr. J.M. Cook). A further addition of 0.5 μM aliquot of the imidazopyridine was required to forestall the low and very low affinity sites so that only high affinity sites are available [21]. Adjacent slices were incubated with 50 mM Tris HCl in presence of [^3^H] Ro 15-1788 ± 20 mM flunitrazepam for the determination of non-specific binding that varied from 20% to 60% of total binding. After drying, slides were apposed to a [^3^H]-sensitive Hyperfilms (Amersham, Italy) for 10 days, the films was developed and autoradiograms were captured via a Panasonic Telecamera (Canon Objective Lens FD 50 mm, 1:3.5). Densitometric quantification was handled using a computer-assisted image analyzer system by running a National Institute of Health Image software (Scion-Image 2.0).

### RT-PCR and in situ hybridization assay

Total RNA was extracted from the entire brain of *Syrian golden hamsters *(n = 3) by using TRI reagent (Sigma, Italy) dissolved in DEPC-water (Sigma, Italy) as previously reported [22]. The integrity of RNA was established by its fractionation on 0.8% agarose gel and staining with ethidium bromide. Total RNA concentrations were determined using a NanoDrop ND-1000 spectrophotometer (NanoDrop Technologies, USA). Isolated RNA was finally frozen at -80°C until further processing. Briefly, reverse transcription reaction (RT) was performed using 2 μg of total RNA according to High Capacity cDNA Reverse Transcription Kit (Applied Biosystem, Italy). Polymerase chain reaction (PCR) using Taq Polymerase (Promega, Italy) was handled for all GABA_A_R subunits considered in the present investigation α_1,2,4,5_, β_2,3 _and γ_2,3_. PCR primers specific for each GABA_A_R receptor subunit gene were designed using Beacon Designer software (Bio-Rad Inc., USA) and their specificity confirmed by homology analysis. The thermal cycle conditions for all GABA_A_Rα subunits were as follows: denaturation at 94°C for 3 min plus 35 cycles consisting of denaturation at 94°C for 50s, annealing at a different temperature (57°C for α_1, _α_2 _and α_5; _58°C for α_4_) for 50s and extension at 72°C for 20s, plus final extension at 72°C for 5 min. For both β and γ subunits, 35 cycles of amplification were used with exception of annealing temperatures (53°C for γ_3_, 54°C for β_2 _and 56°C for β_3 _and γ_2_) and subsequently PCR products were purified using a Wizard Kit (Promega, Italy) and processed for sequence reactions (BMR genomics, Italy).

To perform *in situ *hybridization, antisense and sense probes for each subunit were designed on the basis of the partial sequences obtained in our rodent model and labeled by 3'-tailing using digoxigenin-11-dUTP (DIG) according to the indications supplied by DIG oligonucleotide tailing kit (Roche, Italy). The preparation of the probe was done via its incubation at 37°C for 30 min and then stopped with 0.2 M EDTA pH 8.0. Probe concentration was determined by its quantification against known standards on Hybond N^+ ^filters (Amersham, Italy). Afterwards, brain sections (10 μm) of NHIB and HIB animals, which were previously mounted on polylysine coated slides (Carlo Erba, Italy) and stored at -40°C, were incubated with 100 ng of antisense probe in 100 μl of hybridization solution for overnight incubation at 50°C in a humidified chamber [23]. Nonspecific hybridization was obtained on slides incubated with the sense probe. For immunological detection, sections were coverslipped for 45 min with PBS buffer containing 2% normal sheep serum (Sigma, Italy) and 0.3% Triton X100 (Sigma, Italy). Then an anti-digoxigenin alkaline phosphatase antibody (Roche, Italy) 1:100 was added for 2 h at room temperature and the alkaline phosphatase color reaction buffer (NBT/BCIP) was added to sections and incubated for 72 h in a humidified dark chamber. Neuronal hybridization signals were observed at a bright-field Dialux EB 20 microscope (Leitz) under a phase contrast objective (×40) and transcriptional activity was evaluated with a Panasonic Telecamera (Canon Objective Lens FD 50 mm, 1:3.5) attached to a Macintosh computer-assisted image analyzer system running an Image software of National Institutes of Health (Scion-Image 2.0) plus a constructed internal standard curve for calibrating optical density (O.D.) values. The different hypothalamic nuclei were identified on some cresyl violet stained sections using the hamster atlas [24] so that it was possible to evaluate their O.D. densities.

### Statistical analysis

The expression levels of the major GABA_A_R α, β and γ subunits in some hypothalamic areas of HIB and NHIB hamsters were determined by a two-way Analysis Of Variance (ANOVA) followed by a *post hoc *multiple range Newman-Keul's test when *p*-value ≤ 0.05. As for the establishment of the predominant α subunits expression percentage in these two physiological states, transcript levels of single subunits with respect to total α subunits considered in this study were determined by using a one-way ANOVA followed by a Newman-Keul's multiple range *post hoc *test when a significant *p*-value ≤ 0.05.

## Results

### Competition binding study

**Figure 1:**
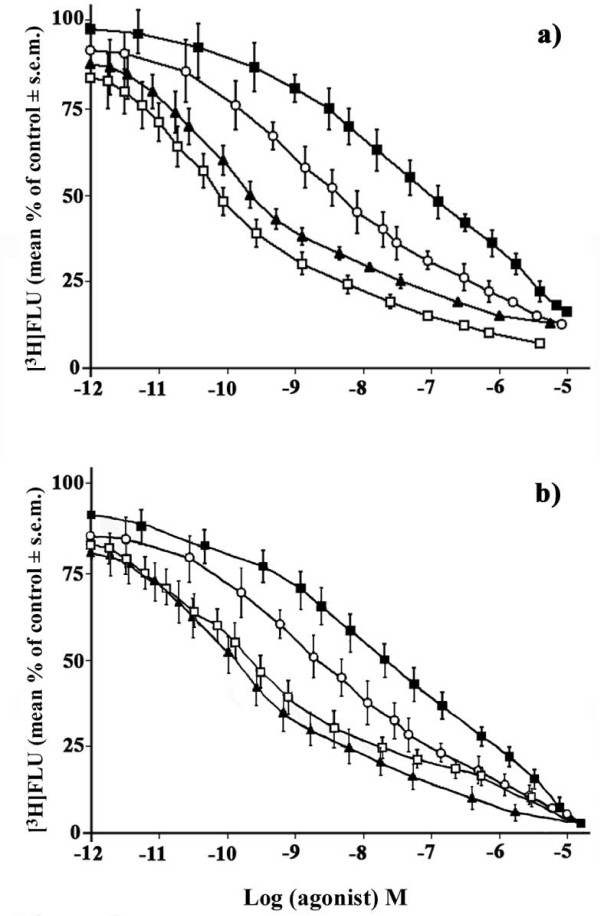
**Competition curves of [^3^H] Ro 15-1788 in the Sch of HIB and NHIB *hamsters***. Displacement curves of [^3^H] Ro 15-1788 (mean % of total binding ± s.e.m) showing the differing binding capacities in the suprachiasmatic nucleus (Sch) of a) HIB and b) NHIB hamsters. Competition study was carried out in the presence of different concentration (500 nM-1 nM) of α_1 _(zolpidem, white square) and α_2 _(flunitrazepam, black triangle) agonists plus α_4 _antagonist (Ro 15-4513, black square) and α_5 _inverse agonist, (Ry 080, white circle) as described in "Materials and Methods". Each point represents the mean of five separate tests.

In the present it was our intention to identify and establish the order of specific α-containing neuronal fields on the basis of their affinity levels characterizing some of the major hypothalamic areas during either HIB or NHIB states of our hamster model. Indeed, the labeling of the different hypothalamic sections with the radioligand [^3^H] Ro 15-1788 in the presence of distinct α subunit drugs and namely α_1 _(zolpidem) and α_2 _(flunitrazepam) agonists, plus α_4 _(Ro 15-4513) antagonist as well as a α_5 _(Ry 080) inverse agonist supplied a heterogeneous distribution pattern. In particular the results of the preliminary study, which confirmed previously published results [25], tended to point out that it was mainly α_1 _and α_2 _subunits of HIB (Figure 1a) and NHIB (Figure 1b), respectively, supplying greater high affinity type of characteristic as shown by their varying binding affinities and Bmax going from a high affinity range of 9.31 × 10^-2 ^nM (327 fmol/mg protein) to 2.47 × 10^-1 ^nM (404 fmol/mg protein) for these corresponding subunits. Such a relationship was also characterized by lower type of binding affinities of α_4_-containing sites and precisely 51.67 nM (Bmax = 215 fmol/mg protein) for NHIB hamsters while an affinity of 356.13 nM (Bmax = 393 fmol/mg protein) was obtained for hibernators (check their order in Figures 1a,b).

### GABA_A_R Molecular Analysis and hypothalamic α subunit expression

On the basis of the aforementioned considerations, our attention was directed towards the distribution and expression pattern of the major GABA_A_R subunits and namely α_1,2,4,5_, β_2,3 _and γ_2,3_. Application of specific primers designed on highly conserved regions of mammalian GABA_A_R subunit mRNAs allowed us to obtained a single cds fragment of 73 bp for GABA_A_R α_1 _[GenBank accession no. 1304461], 90 bp for GABA_A_R α_2 _[GenBank accession no. 1300238], 157 bp for GABA_A_R α_4 _[GenBank accession no. 1300240], 80 bp for GABA_A_R α_5 _[GenBank accession no. 1300246], 68 bp for GABA_A_R β_2 _[GenBank accession no. 1300230], 62 bp for GABA_A_R β_3 _[GenBank accession no. 1304467], 129 bp for GABA_A_R γ_2 _[GenBank accession no. 1300208], 145 bp for GABA_A_R γ_3 _[GenBank accession no. 1304463] which is specific for *Mesocricetus auratus *(Table 1). The partial sequence of GABA_A_R α_1,2,4 _subunits showed a homology >94% and >83% to cds sequences of *Rattus norvegicus *and *Mus musculus*, respectively, whereas GABA_A_R α_1,2,4 _showed a homology of 79% to cds sequence of both *Rattus norvegicus *and *Mus musculus*. Similarly, β_2,3 _subunits showed a homology >93% to cds sequences of both *Rattus norvegicus *and *Mus musculus *whereas γ_2,3 _subunits showed an alignment that is well fitted (> 84%) to cds sequences of these two rodents.

**Table 1:** Primer sequences for the different genes studied

Gene	Forward Primer (5'-3')		Reverse Primer (5'-3')
α_1_	AAAGTGCGACCATAGAACCGAAAG		GCGGAAAGGCTATTCTTGACAGTC
α_2_	GGACGGGAAGAGTGTAGTCAATG		TTTGGAAATGGTAGAGAGGACAGG
α_4_	AGCAGCAAGAGGTCTTTCGTC		AGAAGGTGGTGGAGCAGAGG
α_5_	CCATCCTCCAAACATCCCAAG		CGATCTTGCTGATGCTGCTGCAGG
β_2_	AACTACATCTTCTTTGGGAGAGG		GGTCCATCTTGTTGACATCCAG
β_3_	ACAACTCAGGAATCCAGTATAGG		CCGTAGGTGCGTCTTCTTG
γ_2_	CAACAGGATGCTGAGAATTTGGAATG		GCTGTGACATAGGAGACCTTGGG
γ_3_	ATCACCACACCCAACCAG		CGTCCAACGATAAATCATTTCTTC

**Figure 2:**
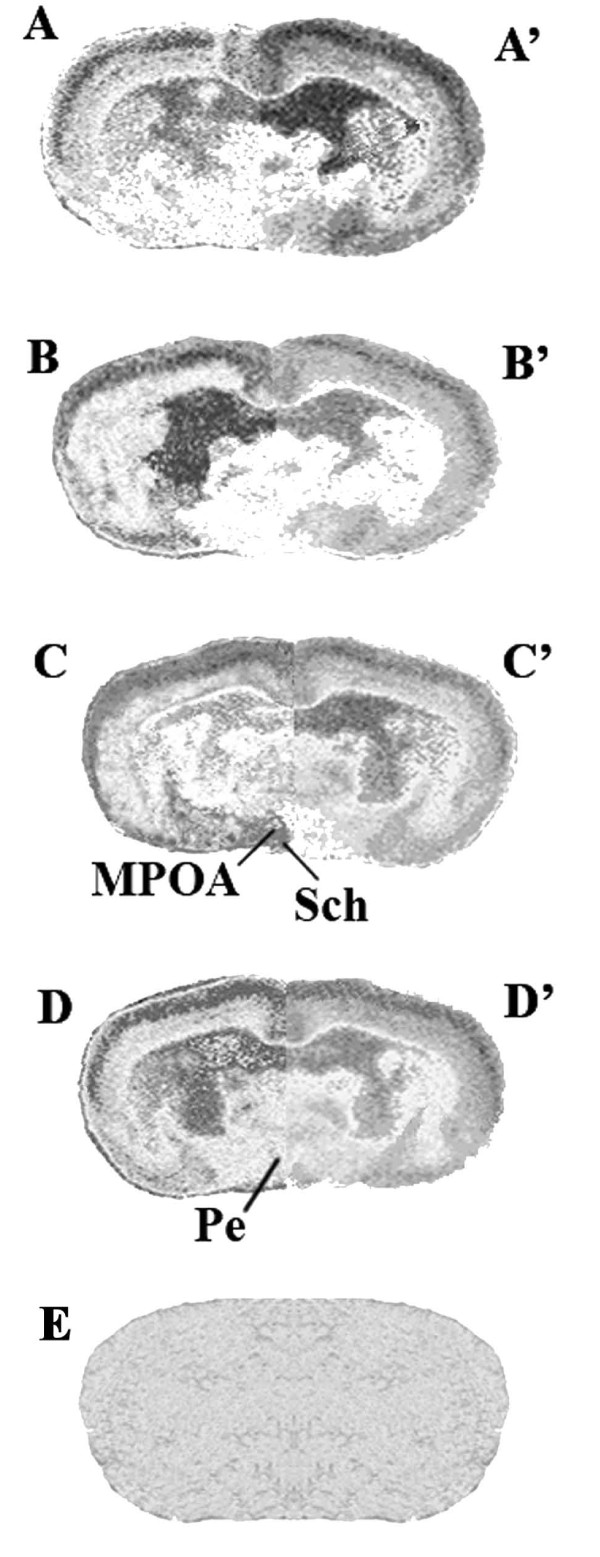
**Representative autoradiograms of hypothalamic areas of HIB hamster**. Well integral representative transverse sections of hypothalamic areas were used to determine the expression pattern of the α GABA_A_R subunits considered in the present study. For this purpose oligonucleotidic antisense for α_1 _(A), α_2 _(B), α_4 _(C), and α_5 _(D) mRNAs developed in the brain of HIB *Mesocricetus auratus *and was compared to the same subunits (A', B', C', D') for NHIB hamsters with respect to (E) nonspecific binding autoradiograms. In this case the nonspecific binding section of HIB proved to be similar to NHIB and so was used for all determinations. MPOA: medial preoptic area; Pe: periventricolar hypothalamic nucleus; Sch: suprachiasmatic nucleus.

**Figure 3:**
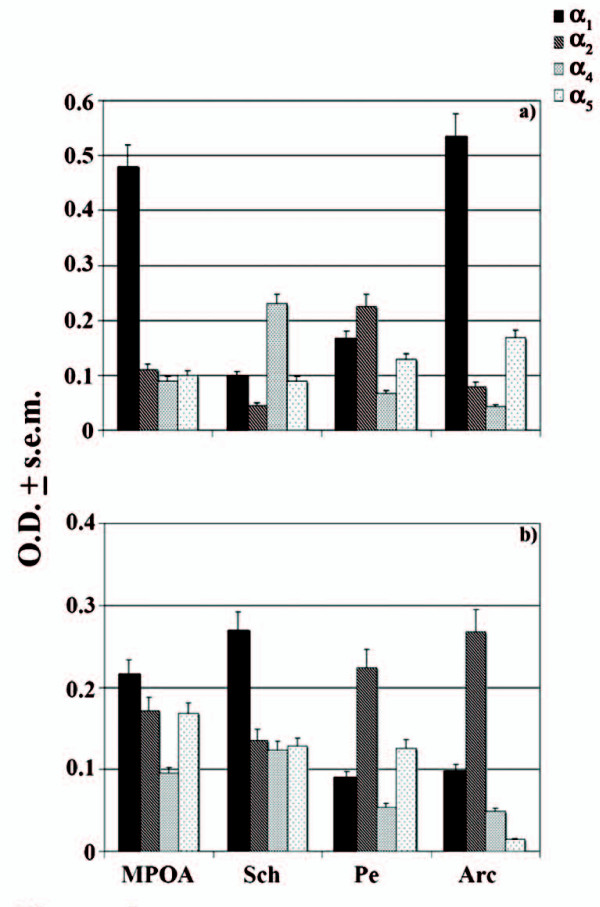
**O.D. of α_1,2,4,5 _mRNA GABA_A_R expression in hypothalamic areas of NHIB and HIB hamsters**. Expression pattern (O.D. ± s.e.m.) of α_1,5 _mRNA GABA_A_R in NHIB (a) and HIB hamsters (b). Arc: arcuate hypothalamic nucleus; MPOA: medial preoptic area; Pe: periventricolar hypothalamic nucleus; Sch: suprachiasmatic nucleus.

Once synthesized, these GABA_A_R α subunit sequences supplied us with a net heterogeneous distribution pattern of the different α-containing receptors in the above hypothalamic neurons as indicated by low, intermediate and high expression levels in a representative autoradiogram of hypothalamic areas for α_1 _(Figures 2A, A'), α_2 _(Figures 2B, B'), α_4 _(Figures 2C, C') and α_5 _(Figures 2D, D') subunits with respect to nonspecific binding levels (Figure 2E) of both HIB (A-D) and NHIB (A'-D') hamsters. In the first case, elevated O.D. expression signals (> 0.40 O.D.) of α_1 _(Figure 3a) were reported to be typical of the medial preoptic area (MPOA) and arcuate nucleus (Arc) in NHIB hamsters while an intermediate level (< 0.4 > 0.18 O.D.) was instead detected for α_2 _of the periventricular nucleus (Pe) under the same physiological state. As far as HIB hamsters are concerned (Figure 3b), intermediate levels of α_1 _and α_2 _were reported for both the suprachiasmatic nucleus (Sch) and Arc, resepctively. Conversely the expression of the other two α subunits were either of an intermediate level as in the case of HIB hamsters or of a very low nature (< 0.180 O.D.) for NHIB animals.

**Figure 4:**
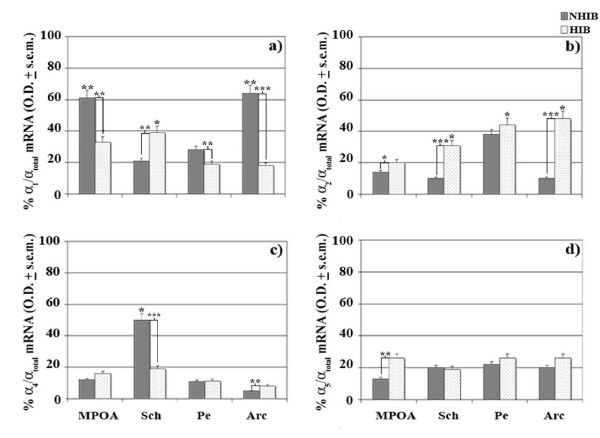
**Percentage of α_1,2,4,5 _mRNA GABA_A_R expression in hypothalamic areas of NHIB and HIB hamsters**. % differences of α_1 _(a), α_2 _(b), α_4 _(c) and α_5 _(d) expression levels over total α levels in hypothalamic HIB areas were evaluated with respect to their controls and compared to the % differences of NHIB. a,*p < 0.05; b,**p < 0.01; c,***p < 0.001. Arc: arcuate hypothalamic nucleus; MPOA: medial preoptic area; Pe: periventricolar hypothalamic nucleus; Sch: suprachiasmatic nucleus.

Surprisingly, however, when the different levels of the single α subunit were reported as a ratio with respect to total α subunit levels considered in the present study, a peculiarly interesting expression pattern was highlighted in these same hypothalamic areas. First of all, the distribution pattern of the different GABA_A_R subunits (α_1,2,4,5_) notably differed in the hypothalamic areas of NHIBs as displayed by a very strong (p < 0.001) up-regulation of the α_1 _subunit (80%) in MPOA during such a physiological state with respect to HIB animals (Figure 4a). Contextually, the other hypothalamic areas of NHIB continued to maintain notably high expression capacities of this specific subunit as shown by very strong and moderately higher (p < 0.05) levels in Arc and Pe, respectively, while very strong levels, instead, characterized Sch of HIBs. On the other hand, elevated α_2_-expressing neurons seemed to be featured in almost all hypothalamic areas of HIBs as shown by a very strong up-regulated expression pattern in Sch and Arc while only a moderate increase was detected in MPOA (Figure 4b). As far as α_4 _subunit is concerned, a very strong up-regulation seemed to be mostly featured in Sch of NHIBs whereas a strong increase was reported for Arc of HIBs (Figure 4c). Nonetheless, α_5_-expressing neurons did not show any evident variations during hibernation with the exception of a somewhat strong up-regulation in MPOA (Figure 4d).

**Figure 5:**
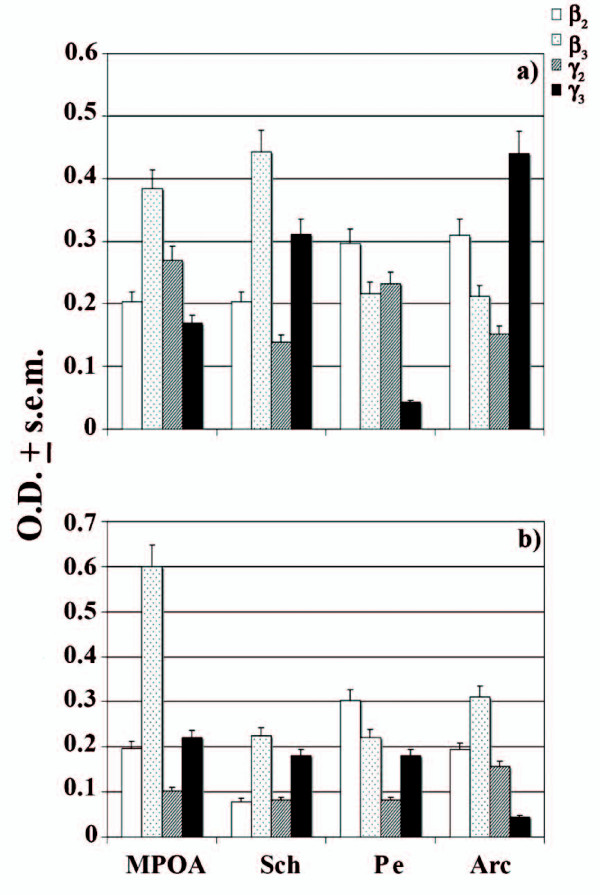
**β_2,3 _and γ_2,3 _levels (O.D. ± s.e.m.) in NHIB and HIB hypothalamic areas**. Expression pattern (O.D. ± s.e.m.) of GABA_A_R β_2,3 _and γ_2,3 _mRNAs in a) NHIB and b) HIB hamsters. Statistics: ANOVA and Neuman Keul's test, * p < 0.05; ** p < 0.01; *** p < 0.001. Arc: arcuate hypothalamic nucleus; MPOA: medial preoptic area; Pe: periventricolar hypothalamic nucleus; Sch: suprachiasmatic nucleus.

A similar trend to that of α subunits was also established for β- and γ-containing neurons in the same hypothalamic areas of NHIB and HIB states. In particular, intermediate levels (< 0.4 > 0.18 O.D) of β_2 _were reported for Pe and Arc of NHIBs, whereas elevated expression signals (> 0.40 O.D.) were obtained for β_3 _subunit in Sch and MPOA of the same physiological state (Figure 5a). Curiously in the case of HIBs (Figure 5b), elevated levels of β_3 _were detected in MPOA, while intermediate levels were typical of Arc and Sch. Regarding γ subunit, it was γ_3 _that showed very strong densities in Sch and Arc of NHIBs whereas in the case of γ_2_, only intermediate levels were observed in MPOA and Pe. However, it was still MPOA that maintained notably moderate levels of γ_3 _in HIBs while this subunit is weakly expressed (< 0.180 O.D.) in Pe and Sch during this same physiological state.

## Discussion

The results of this work highlighted the participation of distinct hypothalamic α GABA_A_R containing neurons during the different HIB bouts of the Syrian golden hamster. In order to determine which specific α subunit was involved in such a physiological state, it was necessary to evaluate the type of binding affinities of α agonists and antagonists that were related to hibernation. Their highly selective inhibiting binding profiles of the different subunit drugs and precisely α_1 _(zolpidem), α_2 _(Flu), α_4 _(Ro 15-4513), α_5 _(RY 080) showed that these agonists bind tightly to most α GABA_A_R containing brain sites in a similar heterogeneous manner to that of rats as well as to that of early appearing HIB mammals such as the hedgehog [21]. Even from the binding differences detected in the present study, it appeared that α_1 _subunit in particular bound to its site at a greater affinity in mainly telencephalic areas [26] suggesting that this specific subunit may be a key neuronal regulating element at least during the different HIB states of rodents.

It was interesting to note that the expression pattern of all α GABA_A_R subunits considered, using specific α_1,2,4,5 _cDNA probes sequenced for *Mesocricetus auratus*, confirmed previously obtained binding trends of the selective α agonists and antagonists [21]. In the first place α_1 _continues to be the major subunit even in most hypothalamic areas as shown by very strong and strong high levels in MPOA and Arc, respectively, of NHIBs and this should not surprise us since such a GABA_A_R subunit has proven to be essential in energy balance- and reproductive activity-controlling site such as MPOA and Arc during hibernation [27]. On the other hand, α_1 _expressing neurons supplying moderately high levels in Sch of HIBs tend to corroborate homeostatic related effects especially during the transition from an awakening to a torpor state with the consequent induction of non-rapid eye movement (NREM) sleep [28]. Indeed during the arousal state, the switching ON of α_1 _may lead to a structurally well-assembled GABA_A_R complex [29] and consequently the activation of motor-controlling neurogenic programs in order to face new functional plasticity states [30]. Moreover, the predominance of a α_1_-dependent pharmacological organizational and functional features [8] have already been reflected during the early neuronal developmental stages of another major limbic region in hamsters and precisely the hippocampus [31] as well as on the induction of visual functions in other adult rodents [32]. As a consequence, it might very well be that the high levels of hypothalamic α_1_-containing neurons may assure a pharmacological protective role against ischemic insults during the awakening phase [19,33] especially since an increased gene expression of this subunit has been correlated to the new functional plasticity states during the arousal phase [34].

Regarding α_2 _and α_5_, these subunits were largely expressed in Arc, Pe plus in Sch, Pe and MPOA, respectively, of mainly HIBs. The lack of any evident variations of the latter subunit in almost all hypothalamic areas, aside that of MPOA, in HIBs and NHIBs tends to represent a constant presence in all facets of the animal's physiological conditions, since α_5 _has proven to play a major role on the activation of distinct GABA_A_R pharmacological kinetic properties throughout the various biological developmental stages [35]. Conversely, the detection of prevalently elevated α_2 _levels in HIBs appears to support a compartmentalized type of inhibitory activity during this physiological state. It is especially during this condition that some vital neuroendocrine functions are changing and so α_2 _could very likely lead to the activation of the arousal state via the induction of these vital functions and namely feeding, which has been shown to be related to altered levels of α subunits [36].

Of particular interest is the dense expression of α_4 _in Sch of euthermics and this tends to support a major role played by the α_4_-containing GABA_A_Rs in such a circadian center [37]. Now the fact that low expression levels of this subunit was detected in the key hypothalamic circadian center tend to underlie a switching ON of homeostatic neuronal processes, which in turn may be linked to awakening states and thus strengthening the importance of specific α_4 _agonists, such as gaboxadol during insomnia bouts [28]. In this context, Sch α_4_-containing GABA_A_Rs may be viewed as major elements for the registering of metabolic [7] and temperature sensitive neuronal changes during thermoregulation and sleep-wake control in a similar manner to that of MPOA and of diagonal band of Broca in other rodents [38,39].

Similarly to the α subunits, even the β- and γ-containing GABA_A_Rs displayed a heterogeneous distribution pattern in most hypothalamic areas and this confirms the major role played by the three subunits throughout the entire mammalian phylogeny [8,40]. β_3 _proved to be a first subunit that showed evident variations in not only Sch but also in MPOA neuronal fields of euthermics; a relationship that tends to point out the major role of β_3 _during the awakening stages of hibernation since this subunit has been shown to be involved numerous homeostatic events, above which the modification of thermoregulatory responses [41-43] that are known be vital for hibernators [5]. Even in this case high expression levels of MPOA β_3_-containing neurons appear to constitute a major neuroprotective element during the arousal states [44] in a comparable manner to its role on homeostatic conditions including body weight, sedative events [14,45] and overall wakening states [46]. Furthermore, the importance of this subunit is supported by knockout mice displaying a key regulatory role, aside that related to developmental and body weight, on the modification of the different forms of sleeping states [47] including anesthesia [14]. The prevalence of elevated β_2_-expressing neurons in most hypothalamic areas during both euthermia and torpor states should not be so surprising since this subunit comprises at least 50% of GABA_A_Rs in the various brain regions [48] as well as being a key constituent of some major neuroendocrine or circadian events [49]. In the case of the other class of GABA_A_R subunits (γ), it appears that the prevalent expression of γ_2 _occurring mostly in MPOA and Pe of NHIB hamsters and this could very well represent a critical condition for synaptic clustering of the GABA_A_Rs with consequently physiologically adequate inhibitory signals at least during the various motor activities [35,50,51]. In a similar manner to the other subunits, a predominantly elevated expression pattern of γ_3 _was also featured in hypothalamic areas such as Sch and Arc of NHIBs along with a comparable condition being detected in the former hypothalamic area plus MPOA and Pe of HIBs. Interestingly, the predominance of γ_3 _during both physiological states seems to underlie the major role elicited by this subunit γ_3_, which seems to fit well with the early and correct assembly of the other synaptic-containing γ subunits required for neuronal trafficking strategies of the various brain regions [52].

## Conclusions

The results of the present study seem to point to a preferential role of the different αβγ subunits in some hypothalamic areas during the different HIB states of the hamster. In particular, the predominantly dense levels of these major subunits permitted us to assign, for the first time, specific subunit triplets to single hypothalamic nuclei and precisely α_1_β_3_γ_2 _in MPOA and α_4_β_3_γ_3 _in Sch of euthermics while α_2_β_3_γ_2 _appears to be typical of Arc in the HIBs. We are still at the beginning but the identification of a brain regional distribution pattern of distinct GABA_A_R subunit combinations operating during hibernation may have interesting bearings on the development of new therapeutic approaches for neurological disorders. In this case the identification of α-containing brain regions cross-talking with other major neuroreceptor systems such as orexinergic enriched brain regions [36] may very well supply interesting insights regarding ischemic conditions during arousal states of HIBs [19], or insomnia conditions linked to hippocampal cAMP-dependent signaling alterations [53].

## Abbreviations

GABA_A_R: γ-aminobutyric acid type A receptor; NHIB: non-hibernating hamsters; HIB: hibernating hamsters; MPOA: medial preoptic area; ARC: arcuate nucleus; PE: periventricular nucleus; SCH: suprachiasmatic nucleus; BDZ: benzodiazepines; RT-PCR: reverse transcription reaction-Polymerase chain reaction; DEPC: diethylpyrocarbonate; DIG: digoxigenin-11-dUTP; EDTA: ethylene diamine tetraacetic acid; PBS: phosphate buffer solution; NBT/BCIP: alkaline phosphatase color reaction buffer; O.D.: optical density; NREM: non-rapid eye movement.

## Competing interests

The authors declare that they have no competing interests.

## Authors' contributions

RA, RMF and MC conceived, designed the experiments, wrote and edited the manuscript. AE carried out *in vitro *quantitative autoradiography and *in situ *hybridization. DVA developed and performed RT-PCR experiments. CA performed the statistical analysis. All authors participated in analysis of dates, read and approved the final manuscript.
